# Increase in Phloem Area in the Tomato *hawaiian skirt* Mutant Is Associated with Enhanced Sugar Transport

**DOI:** 10.3390/genes12060932

**Published:** 2021-06-18

**Authors:** Fabien Lombardo, Pietro Gramazio, Hiroshi Ezura

**Affiliations:** 1 Faculty of Life and Environmental Sciences, University of Tsukuba, Tsukuba 305-8572, Japan; piegra@upv.es; 2 Tsukuba Plant Innovation Research Center, University of Tsukuba, Tsukuba 305-8572, Japan; ezura.hiroshi.fa@u.tsukuba.ac.jp

**Keywords:** pedicel, vascular tissues, phloem, carbon partitioning, tomato

## Abstract

The *HAWAIIAN SKIRT* (*HWS*) gene has been described in Arabidopsis, rice, tomato and poplar where it seems to perform distinct functions with relatively little overlap. In tomato, alteration of the gene function confers facultative parthenocarpy, thought to be a consequence of changes in the microRNA metabolism. In the rice mutant, improvement in panicle architecture is associated with an increase in grain yield. Knowing that *hws* tomato fruits show a higher Brix level, it was suspected that vascular bundles might also be altered in this species, in a similar fashion to the rice phenotype. The pedicel structure of the *hws-1* line was therefore examined under the microscope and sugar concentrations from phloem exudate were determined in an enzymatic assay. A distinct increase in the phloem area was observed as well as a higher sugar content in mutant phloem exudates, which is hypothesized to contribute to the high Brix level in the mutant fruits. Furthermore, the described phenotype in this study bridges the gap between Arabidopsis and rice phenotypes, suggesting that the modulation of the microRNA metabolism by *HWS* influences traits of agricultural interest across several species.

## 1. Introduction

Tomato (*Solanum lycopersicum*) is one of the major cultivated crops in the world, with over 180 million tons produced every year [1]. In the face of challenges arising from climate change and the increasing food demand, much effort in tomato research is being spent towards the breeding of stress-tolerant, nutrient-enriched varieties. Parthenocarpy, which is the production of fruits in the absence of ovule fertilization, is of particular interest to breeders. Parthenocarpy gives the double advantage of dispensing with emasculation in high-yield F1 hybrid production and diminishing the threat of heat stress over fruit setting. In this context, the *hawaiian skirt* (*hws*) line was identified in a screen for parthenocarpic mutants of the Micro-Tom cultivar [2]. In addition to the parthenocarpy trait, fruits of the *hws-1* line were also of higher Brix, indicative of a sugar increase.

The *hws* mutant had originally been isolated in 2007 in Arabidopsis (*Arabidopsis thaliana*) from a fast-neutron-mutagenized population in a screen for floral phenotypes [3]. The gene designation originates from the distinct fused-sepal phenotype, somewhat evocative of a Hawaiian skirt. In addition to the floral phenotype, seed size and root growth were also found to be influenced by the gene expression level [3]. Later work showed that accumulation of the microRNA (miRNA) miR164 in the mutant promoted the degradation of the *CUC1* and *CUC2* proteins by post-transcriptional gene silencing, resulting in the characteristic fusion of adjacent sepals [4]. Additional evidence supporting the hypothesis of *HWS* modulating miRNA accumulation were found in suppressor screens of Arabidopsis mutants affected in the miRNA pathway by two different research groups [5,6].

In tomato, accumulation of specific miRNA species in the *slhws-1* mutant as well as floral and leaf phenotypes evocative of the Arabidopsis mutant indicated that the gene function is conserved, at least to some extent, in the two species [2]. Furthermore, the tomato mutant can be phenotypically complemented using the *A. thaliana* coding sequence, confirming the orthology relationship between the two genes [7]. The main phenotypic differences when comparing the mutants in both species arise due to the presence of fleshy fruits in tomato, which allows for the observation of the parthenocarpy phenotype and the elevated fruit sugar content as indicated from Brix measurements [2].

In poplar (*Populus tremula* x *P. alba*), the PtaHWS [8] protein shows a high degree of homology with its Arabidopsis and tomato counterparts and is most likely encoded by an orthologous gene, although the orthology relationship has not been firmly demonstrated. *PtaHWS* is thought to be a central regulator of poplar root proliferation in response to nitrogen starvation. Manipulation of *PtaHWS* expression also results in the variation of miR164 levels [8], in accordance with the results of other studies describing *HWS* as an actor in miRNA metabolism [2,4,5].

In rice (*Oryza sativa*), the two functional orthologs *OsEP3* (*ERECT PANICLE3* [9]; formerly designated *LARGER PANICLE* [10]) and *OsFBK1* [11] have been described for their role in panicle morphology and cell wall thickening, respectively. In the *osep3* mutant, both peduncle diameter and vascular bundle number are significantly increased, although only at limited portions of the panicle [12]. Interestingly, a significantly higher grain yield was measured in *osep3*, attributed to the increase in the number of vascular bundles [10].

The molecular mechanisms by which HWS modulates miRNA levels are still unclear and the different phenotypes observed among orthologous mutants may be resulting from distinct, unrelated functions of the gene. *HWS* encodes an F-box protein, suggesting a function within Skip1-Cullin-F-box (SCF) complexes in protein degradation via the 26S proteasome machinery [13]. The FBK1 protein, encoded by one of the rice *HWS* orthologs, was shown to interact with a reductase involved in lignin synthesis to presumably tag it for degradation [11]. However, no such targets within the miRNA biosynthesis pathway could be identified, despite the efforts of different research groups [5,6,14].

In addition to the extensively described reproductive defects of the *slhws-1* line, a number of morphological alterations in general plant architecture have also been reported [2]. Among them, the stem diameter was measured to be significantly larger in two-month-old mutant plants, evoking the rice phenotype. A visual inspection of the mutant pedicels during all stages of plant development indicated that they were also of a generally larger diameter than their WT counterparts, prompting for a closer examination.

In an effort to understand the *HWS* gene function in a broad context, the present study was aimed at determining whether *slhws-1*, which shares the miRNA accumulation phenotype with Arabidopsis, would also share vascular tissue changes resembling the ones described in the rice *osep3* mutant. The results described later in this study substantiate the hypothesis that *HWS* promotes phloem development, presumably via the accumulation of specific miRNA species, thereby enhancing sugar transport. This attributes a role for *HWS* in carbon partitioning which is likely to be conserved across several species of agricultural interest.

## 2. Materials and Methods

### 2.1. Plant Materials and Growth Conditions

The tomato (*Solanum lycopersicum*) *hws-1* line was isolated from an ethyl methanesulfonate (EMS) mutagenized population in cv. Micro-Tom genetic background [15,16]. The two other alleles *slhws-2* and *slhws-3* reported in [2] produce only few or almost no seeds, respectively, making their study impractical. For this reason, only *slhws-1* was investigated. All experiments were performed on BC3 populations grown in rockwool supplemented with a Ōtsuka house 1 and 2 (OAT Agrio Co., Ltd, Tokyo, Japan) nutrient solution under a 16-h light/8-h dark regime under LED lights outputting 230 μmol m^−2^ s^−1^ of PAR.

### 2.2. Microscopy

Pedicels were cut halfway between the abscission zone and the fruit, corresponding to the pedicel’s smallest diameter point, using two razor blades taped on both sides of a microscope slide. This allowed freehand cutting of reasonably thin sections that were directly stained with 0.05% toluidine blue for approximately 5 minutes. Sections were briefly rinsed with distilled water and observed under an Olympus BX53 light microscope (Olympus Corporation, Tokyo, Japan) using an imaging software (cellSens Standard 1.6, Olympus, http://www.olympus-global.com (accessed on 10 May 2021)). Images were edited for brightness, sharpness and color balance using Adobe Photoshop (Adode Inc., 151 South Almaden Boulevard, San Jose, CA, USA). Size and area measurements of cut biological material were done using the Fiji distribution of the ImageJ software [17,18]. At least 20 pedicels from five individual plants per line were observed.

### 2.3. Phloem Exudate Sugar Quantification

Phloem exudates were collected as described in [19] with minor modifications: PCR tubes filled with 200 μL of EDTA-HEPES buffer solution were used and sealed with parafilm to prevent loss by evaporation. After leaving the pedicels immersed during 7 h of the dark hours, 13 μL were used in a Sucrose/D-Fructose/D-Glucose assay kit (Megazyme, Wicklow, Ireland) and sugars were quantified using a plate reader following the manufacturer’s instructions. Three technical replicates of 26 and 15 biological replicates (WT, *hws-1*, resp.) were measured. The quantity of the remaining buffer after collection was measured by weighing the liquid with a precision scale.

### 2.4. Photosynthetic Assimilation Measurements

The light-response curves were determined using an open-flow infrared gas exchange analyzer system (Li-6400XT, Li-Cor, 4647 Superior Street, Lincoln, NE, USA). All measurements were made on fourth terminal leaflets and completely expanded leaves. The analysis was conducted under common conditions with an airflow rate of 500 μmol s^−1^ into a 6 cm^2^ chamber. Leaf area was measured for calculations using ImageJ as described earlier [17,18].

### 2.5. Bioinformatics Analysis

The *HWS* gene is referenced as Solyc01g095370. The sequence of the HWS protein was retrieved from the Sol Genomics Network database (https://solgenomics.net/ (accessed on 3 May 2021)) and a blastp (protein-protein BLAST) with default parameters was performed using the National Center for Biotechnology Information (NCBI; https://blast.ncbi.nlm.nih.gov/Blast.cgi (accessed on 3 May 2021)) database on the non-redundant protein sequences (nr) data subset. From the results obtained, only species of agronomical interest and some of their wild relatives were selected for the protein alignment using the Clustal Omega webtool (https://www.ebi.ac.uk/Tools/msa/clustalo/ (accessed on 3 May 2021)) [20]. The alignment was plotted using the Jalview software [21].

## 3. Results

### 3.1. The Diameter of hws-1 Pedicels Is Larger

**Figure 1:**
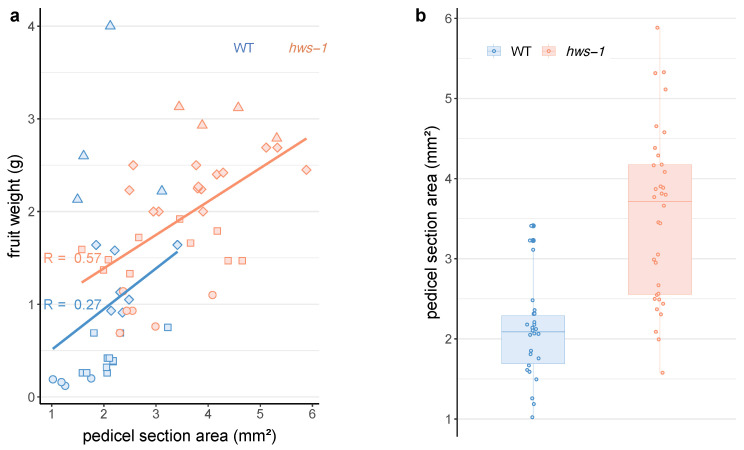
The larger fruits of *hws-1* are associated with wider pedicels. (**a**) Pedicels carrying fruits aged from 7 to 16 DAA (○: 7~9; □: 11~12; ⋄: 13~14; Δ: 15~16) were cut halfway between the fruit and the abscission zone. (**b**) Pedicels of *hws-1* are markedly wider than their WT counterparts, and the differences are statistically significant with p<0.001 in an unpaired *t*-test. In both (**a**,**b**), section areas were measured using the ImageJ software [17,18]; n=60.

To investigate the relation between fruit growth and pedicel width, fruits aged from 7 to 16 days after anthesis (DAA) were weighed and pedicel size, defined by their transverse section area, was measured. Fruits of *hws-1* were of noticeably larger weight, in accordance with previous observations of their rapid early growth (Figure 1a). Expectedly, there were only a moderate and a low linear correlation between pedicel size and fruit weight in the mutant and the WT, repectively (Figure 1a). Nevertheless, the data clearly indicated that the larger mutant fruits were associated with wider pedicels.

Direct comparison of the pedicel section area in both lines shows that mutant pedicels are about twice as wide as the WT ones (Figure 1b). It was hypothesized that the wider pedicels would accommodate for an increased number of vascular elements, most likely contributing to the accelerated growth and higher Brix content of the mutant fruits.

### 3.2. Enhanced Sugar Transport in hws-1

#### 3.2.1. Mutant Pedicels Show Larger Phloem and Narrower Xylem Tissues

**Figure 2:**
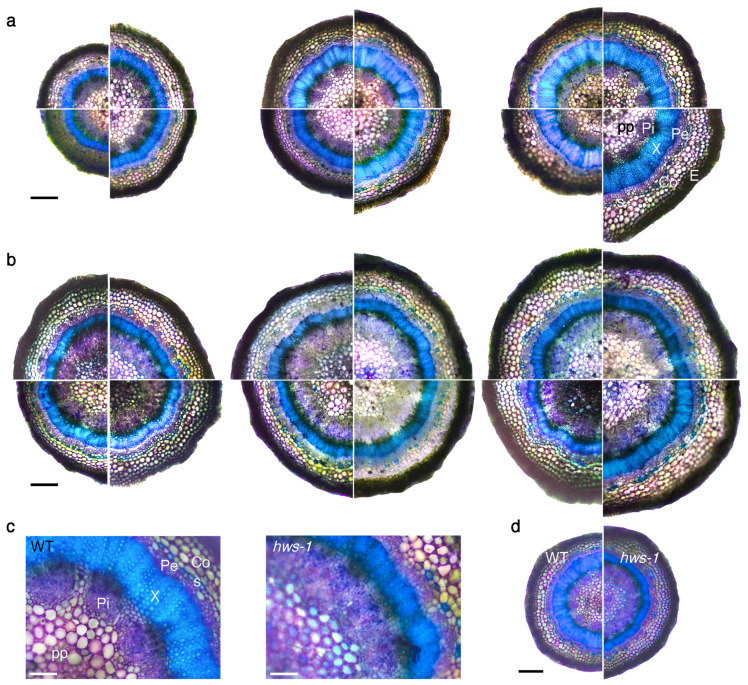
Transverse sections of representative pedicels showing wider phloem and narrower xylem rings in *hws-1*. All sections were stained with toluidine blue prior to light microscopy observation. (**a**) Sections of WT pedicels. Each section is reconstructed from four segments of different pedicels. Segments are shown in a increasing diameter arrangement, clockwise from the bottom left segment. pp, pith parenchyma; Pi, internal phloem; X, xylem; Pe, external phloem; Co, collenchyma; s, sclerenchyma (blue-green stained); E, epidermis. Scale bar represents 250 μm. (**b**) Sections of *hws-1* pedicels, similarly to (**a**). (**c**) Details of selected WT and *hws-1* pedicels with comparable diameters, showing vascular elements of strikingly different sizes. Scale bar represents 100 μm. (**d**) Lower magnification of pedicels in **c**, shown for reference. Scale bar represents 300 μm.

In order to investigate *hws-1* pedicels at the histological level, a series of sections generated from below the abscission zone were observed under the microscope after brief staining with toluidine blue. In the WT, internal phloem elements were spreading radially and disconnecting from each other as pedicel diameter increases, being visibly interrupted by parenchyma cells in larger pedicels, as shown in Figure 2a. The xylem ring, showing a characteristic strong blue staining due to its lignin content, substantially enlarged as pedicel diameter increased. Likewise, the external phloem expanded radially, although with less amplitude, and only the epidermal and the sclerenchyma cell rings maintained a relatively constant size across different pedicel sizes.

In contrast, *hws-1* pedicels displayed a predominantly continuous internal phloem tissue, even in the largest observed pedicels, with only occasional inclusions of parenchymatic cells (Figure 2b). Strikingly, the xylem ring remained narrow across the range of observed pedicels. At equivalent pedicel size, the outer phloem tissue appeared marginally larger in *hws-1*; in contrast, the internal phloem tissue was estimated to be of a total area from 1.3 to 1.8 times larger in the mutant (Figure 2c,d). Considering that mutant pedicels were generally wider (Figure 1b), the average phloem area was deduced to at least twice as large in *hws-1* compared to the WT.

#### 3.2.2. Mutant Phloem Exudates Have a Higher Sugar Content

Having established that the vascular tissue organization is substantially altered in *hws-1*, it was questioned whether sugar delivery to the fruit would also be impacted. Precise estimation of sugar concentrations in the phloem sap is technically challenging, however the EDTA-enhanced exudation technique, which has been used reliably in several species [22], is rather straightforward and allows for a fair estimation of phloem sugar content.

**Figure 3:**
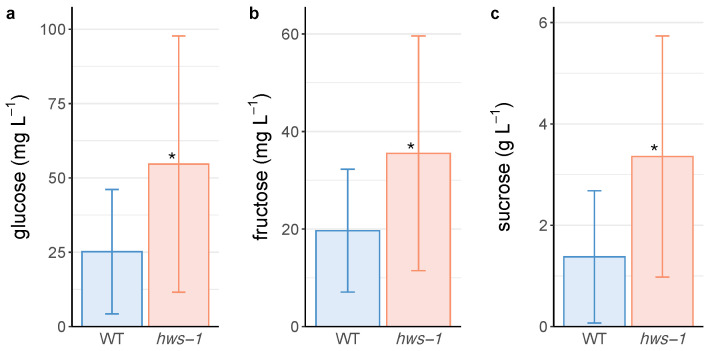
Phloem exudates from *hws-1* contain more sugar than WT ones. Pedicels carrying 8 DAA to 20 DAA-old fruits were cut and phloem exudates were collected for seven of the dark hours in EDTA-HEPES. (**a**) Average glucose concentration in phloem sap exudates (**b**) Similarly to (**a**) for fructose (**c**) Similarly to (**a**) for sucrose. Bars represent mean values with standard deviation. Unpaired *t*-tests indicate significant differences (annotated with * on the graphs) for all three sugars at the p<0.05 level (n=26 and n=15 for WT and *hws-1*, resp.).

To uncover a potential difference in the phloem sugar concentration of *hws-1*, phloem exudates were collected similarly to the method described in [19]. Enzymatic assay of glucose, fructose and sucrose revealed that all three sugars were more concentrated in exudates collected on the mutant. Sucrose was found in a considerably higher amount (Figure 3a) than glucose and fructose (Figure 3b,c), as expected from its role of transport product of the photosynthesis.

Although the concentrations measured in the exudate samples cannot be interpreted as accurate estimations of in vivo phloem sap concentrations since parameters such as phloem flow and phloem pressure turgor have not been taken into account, it is assumed that their statistically significant differences (unpaired *t*-test, p<0.05) reflect an increase in the amount of transported sugar in mutant pedicels.

### 3.3. Carbon Photosynthetic Assimilation in hws-1 Is Comparable with the WT One

The previously reported [2] higher Brix value in *hws-1* fruits may be resulting, partially or entirely, from an increase in source activity. Previous observations of pronounced leaf morphological changes in *hws-1*, namely reduction in leaf serration and fusion of leaflets [2], pointed to an alteration of the photosynthetic potential of the mutant.

To evaluate the stomata distribution in *hws-1*, leaf imprints generated using the nail polish technique were observed under the microscope and stomata density as well as stomata size were determined. Stomata density was found to be comparable with the WT, however stomata were smaller in *hws-1* (Table 1), potentially affecting stomatal conductance.

**Figure 4:**
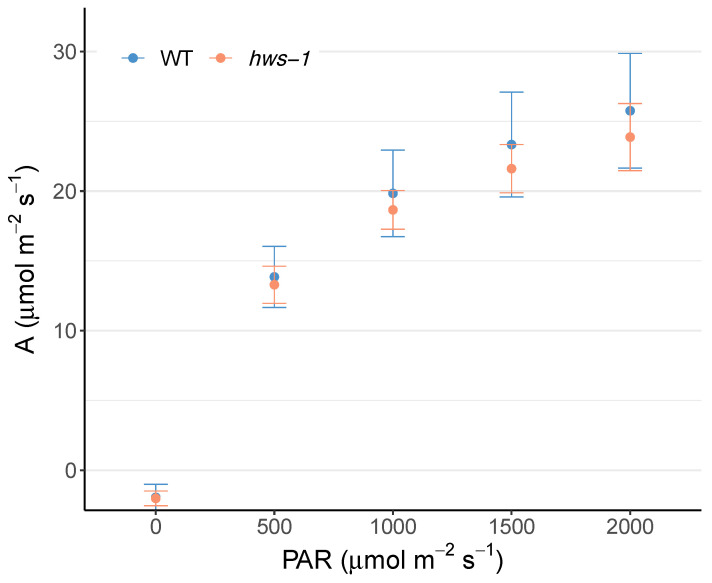
Light-response curves show comparable photosynthetic assimilation between WT and mutant lines. Photosynthetic assimilation was measured on the fourth leaves of 1-month-old plants. Error bars represent standard deviation. Unpaired *t*-tests indicate that difference are not significant at the p<0.05 level (n=8).

To further investigate the photosynthetic potential of the *hws-1* line, a light-response curve was generated in parallel with the WT. Although mean photosynthetic assimilation values were slightly lower for the mutant, they were not statistically different for any of the radiation intensities tested (Figure 4).

The similar photosynthetic assimilation of both lines suggested that enhanced phloem loading, higher sink activity, or a combination of both are responsible for the higher Brix measured in *hws-1* fruits rather than an increased source activity.

## 4. Discussion

In an earlier study [2], the growth rate of *slhws-1* ovaries was reported to be markedly faster than in the WT. Considering that fruits from both lines are of comparable weight when they reach breaker stage, it is assumed that the initial faster growth rate observed in the mutant gradually slows down, allowing for the WT to eventually catch up. Although the development of pedicels cannot reasonably be expected to be linearly correlated all along the fruit development, it is possible that the factors responsible for the rapid early fruit growth in the mutant also promote pedicel growth, accounting for the observed size differences. In normal fruit development, ovary growth is put on hold until fertilization occurs to avoid the unnecessary, resource-costly production of seedless fruits. Upon fertilization, an auxin peak reactivates ovary development, thereby synchronizing both processes [23]. An abnormally high auxin concentration in the ovary can trigger the production of fruits even in the absence of fertilization [24,25]. Several mutants of the auxin pathway show phenotypes remarkably similar to the ones of *slhws-1*. For example, overexpression lines of *SlARF3* results in leaf polarity defects [26] and silenced lines for the *SlIAA9* gene display some degree of leaf and sepal fusion [27]. In this light, the auxin response is most likely altered in *slhws-1*.

Interestingly, the establishment of vascular tissue organization during embryo development is also determined, among other factors, by auxin (reviewed in [28]). The role of auxin has been shown to extend to the determination of the identity of vascular cambium cells [29]. Recently, the characterization of *erecta* mutant lines has underlined the importance of the auxin/cytokinin balance in the formation of vascular patterns [30]. Knowing that many miRNA species accumulate in *hws*, it is hypothesized that auxin signaling is altered in the mutant via the miR390-*TAS3-ARF* pathway [31]. Future investigation of the relation between the miRNA accumulation, auxin signaling and vascular tissue organization should shed light on the molecular mechanisms at play.

The rice *osep3* mutant shows phenotypes strikingly similar to the ones of *slhws-1*. In *osep3*, peduncles are thicker, larger in diameter and the number of vascular bundles significantly increased compared to the WT [12], evocative of the observations in Figure 2. The narrower xylem ring and the larger internal phloem suggest that phloem development occurs to the detriment of xylem development during the secondary growth of *slhws-1* pedicels. As a monocot species, there is no secondary growth in rice and the organization of the vascular tissue is substantially different than in a dicot species such as tomato. Nonetheless, the alterations in the vascularization in both mutants can be regarded as corresponding, suggesting they are resulting from the action of an upstream, common component of the vascular differentiation machinery. Another corresponding feature is the increased grain yield in *osep3* which likens the elevated Brix level of the tomato counterpart. Surprisingly, the photosynthetic activity was found to be reduced in the rice mutant [9]. This decrease was attributed to a lesser stomatal development. No significant differences could be observed for photosynthetic assimilation in our experiment, however the stomatal size was measured to be smaller in *slhws-1*, similarly to the *osep3* phenotype (Table 1, Figure 4). In any case, the hypothesis that an enhanced sugar translocation to the fruits results from an increased photosynthetic activity can be confidently excluded. It is difficult to extrapolate differences of sugar transport efficiency solely based on histological observations since sugar transport is influenced by several physical parameters whose examination was outside the scope of this study [32]. The shorter pedicel length (Table A1) as well as the phloem outgrowth are likely responsible for the enlarged pedicel diameter in *hws-1*. This larger transport area would passively facilitate sugar transport to sink tissues, accounting for the initial faster fruit growth rate in the mutant. Considering that the maximum sugar transport rate is limited by the solution viscosity [33], a larger transport area, rather than an increased phloem loading, would facilitate sugar translocation to the fruits. The higher sugar content for all three investigated sugars in *slhws-1* phloem exudates (Figure 3) supports the hypothesis that the vascular tissue alterations allow for a higher sap flow through the mutant pedicels, contributing to the higher Brix level in *slhws-1* fruits.

**Figure 5:**
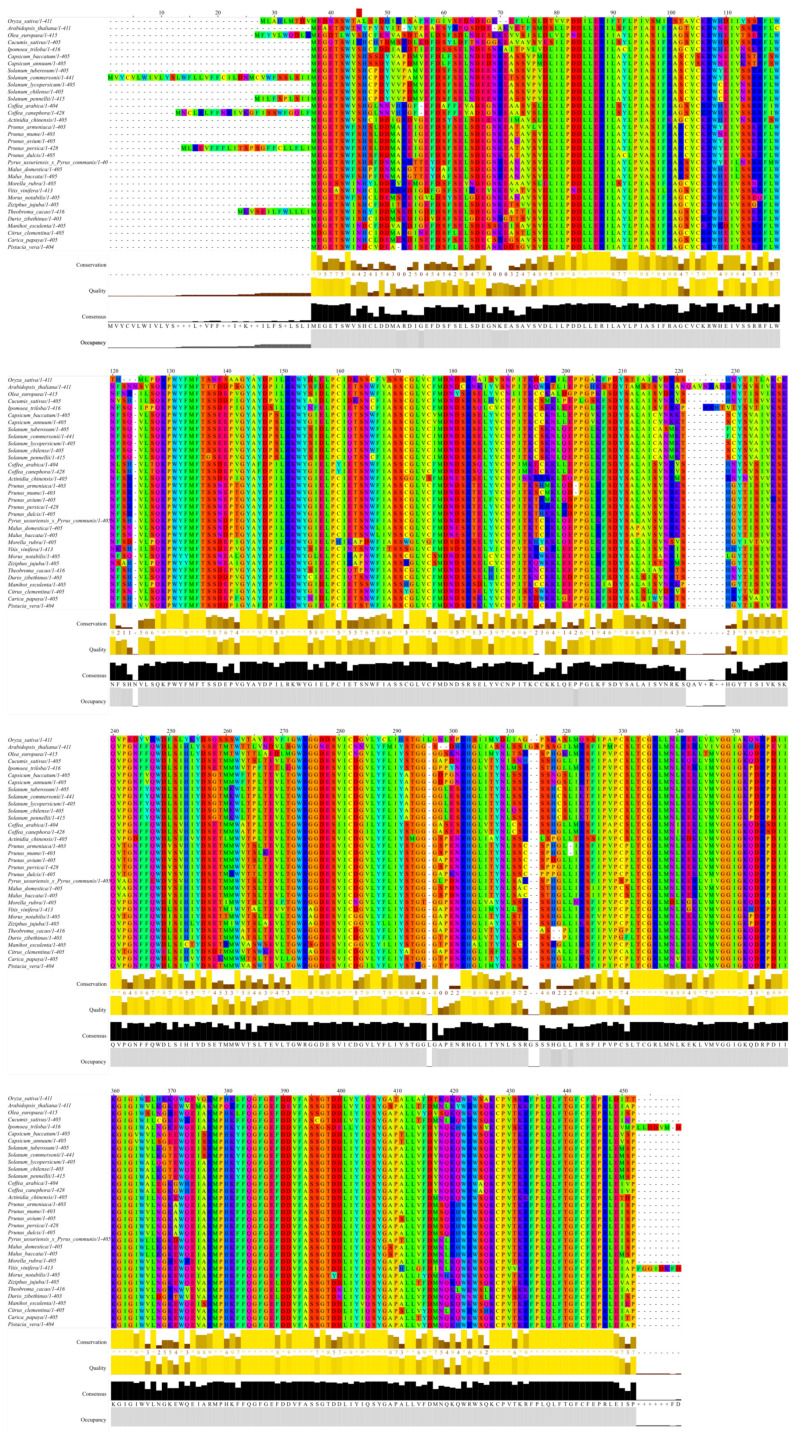
*HWS* orthologs and putative orthologs in crops of interest. The alignement was performed using Clustal Omega [20]. Note that there is some uncertainty about the translational start of the *OsEP3* gene [9]; the sequence displayed here is a truncated version of the originally published one [12].

The *HWS* gene is particularly well conserved in many species of agricultural interest (Figure 5). To our knowledge, there are no reports about changes in the vascular tissue pattern in any of the *athws* mutant described. The occurrence of similar phenotypic traits in *hws* mutants across unrelated species suggests that the gene function is broadly conserved. MicroRNAs have a central regulatory role in plant-environment interactions [34]. Phloem development is tightly linked with plant growth plasticity and is therefore expected to largely be under the control of miRNA species [35]. In this light, the elucidation of the molecular mechanisms by which the *HWS* gene alters vascular tissue organization would provide valuable insights for crop improvement.

## Figures and Tables

**Table 1:** Stomata are smaller in the *hws-1* mutant. Stomata were counted from nail polish imprints from the third, fourth and sixth leaves using the ImageJ software [17,18]. Values represent means with standard deviations given in brackets (n=90 and n=22 for size and density measurements, resp.). Star (${}^{*}$) indicates significant difference at p<0.01 in an unpaired *t*-test.

Line	Stomata Size (μm^2^)	Stomata Density (mm^−2^)
WT	320 (60)	183 (34)
*hws-1*	285 ${}^{*}$ (60)	204 (35)

## Data Availability

Not applicable.

## Abbreviations

The following abbreviations are used in this manuscript:

WT	Wild Type
DAA	Day(s) After Anthesis
PAR	Photosynthetically Active Radiation

## Appendix A

**Table A1:** Pedicel length is shorter in *hws-1*. Pedicel length was measured from the abscission zone to the fruits of 0.8 to 2.5 g (immature green to mature green stages). Values represent means with standard deviations given in brackets (n=20). Star (${}^{*}$) indicates significant difference at p<0.001 in an unpaired *t*-test.

Line	Pedicel Length (mm)
WT	5 (0.6)
*hws-1*	3.1 ${}^{*}$ (0.7)
